# A pilot study: VereMTB detection kit for rapid detection of multidrug-resistant *mycobcterium tuberculosis* in clinical sputum samples

**DOI:** 10.1371/journal.pone.0228312

**Published:** 2020-03-09

**Authors:** Xichao Ou, Qiang Li, Dan Su, Hui Xia, Shengfen Wang, Bing Zhao, Yanlin Zhao

**Affiliations:** 1 National Center for Tuberculosis Control and Prevention, Chinese Center for Disease Control and Prevention, Beijing, P. R. China; 2 Science and Education Department, Beijing Physical Examination Center, Beijing, P. R. China; 3 Department of Pathology, Capital Medical University Affiliated Beijing Chest Hospital, Beijing, P. R. China; The University of Hong Kong, CHINA

## Abstract

The performance of VereMTB^TM^ Detection Kit for detection of multidrug-resistant tuberculosis (MDR-TB) was validated by using 124 sputum samples. Comparing with MGIT culture, the sensitivity and specificity of VereMTB Detection Kit for MTBC detection were 97.0% and 98.3%, respectively. Compared with MGIT DST, the sensitivity and specificity of VereMTB Detection Kit for RIF resistance detection were 85.7% and 93.9%, respectively, and the sensitivity and specificity of VereMTB Detection Kit for INH resistance detection were 75.0% and 95.7%, respectively. 6 NTM samples were also detected and identified correctly. The VereMTB Detection Kit can detect MDR-TB rapidly and accurately in sputum samples from TB suspects.

## Introduction

Tuberculosis (TB) is a respiratory infectious disease that seriously endangers human health. According to the World Health Organization (WHO), 10.0 million new pulmonary TB cases and 1.3 million deaths due to the disease were reported worldwide in 2017 [1]. There are large gaps in the detection and treatment of multidrug-resistant TB (MDR-TB)/rifampicin-resistant TB (RR-TB). In 2017, appropriate treatment was administered for only 25% of the estimated 558 000 MDR/RR-TB cases [1].

Conventional drug susceptibility testing is commonly used for detecting drug-resistant TB, however, this method is time-consuming that requires 8–12 weeks to generate results. Leveraging on PCR technology, relatively faster turnaround time of just a few hours can be achieved in the form of nucleic acid amplification test (NAAT). NAAT can also identify specific molecular markers related to drug resistance thus providing another layer of information for successful patient management [2]. Therefore, rapid detection and retrieving of molecular information of MDR-TB is essential for appropriate treatment and care. Thus, reducing morbidity, mortality, transmission of infection and the emergence of extensively drug-resistant TB (XDR-TB).

VereMTB^TM^ Detection Kit is a NAAT Lab-on-Chip (LoC) assay used for detection and identification of MTBC and nontuberculous Mycobacterium (NTM) as well as the diagnosis of MDR-TB **[3, 4].** Drug susceptibility to RIF and INH is determined together with MTBC detection in a single sourced sample. The workflow comprises three main steps: PCR, Microarray Hybridization and Microarray Analysis. The initial version of this detection kit was previously evaluated and has shown to be user-friendly and fast with good diagnostic performance [4]. In this current version some primers and probes are redesigned to improve the sensitivity for drug susceptibility detection and NTM identification. The current VereMTB Detection Kit is able to identify and differentiate Mycobacterium species- MTBC, (*Mycobacterium abscessus (M*. *abscessus)*, *M*. *chelonae*)*, *M*. *avium*, *(M*. *intracellulare*, *M*. *vulneris*, *M*. *chimaera*, *M*. colombiense)*, (*M*. *simiae*, *M*. *kansasii*, *M*. *scrofulaceum*,*M*. *gastri*, *M*. *mantenii*)*, *M*. *xenopi and M*. *fortuitum* (*unable to differentiate the species in parentheses due to sequence similarity). *IS6110* and *16srRNA* sequences were used for detection and identification of MTBC and NTM respectively. Probes for detecting DST (RIF and INH resistance) remain unchanged-Wild-type *rpoB* hot-spot (codons 510–513, 515–518, 523–526, 530–533), *inhA* (-21/-7) and *katG* (codon 313–317); as well as mutations L511P (ctg/ccg), D516V (gac/gtc), H526D (cac/gac), H526Y (cac/tac), S531L (tcg/ttg) for *rpoB*, t-8a, t-8c, c-15t for *inhA*, and S315T (agc/acc, agc/aca) for *katG*. In this study, we evaluated the diagnostic performance and the feasibility of this current version of VereMTB Detection Kit using sputum samples for MTBC/ NTM and MDR-TB detection.

## Materials and methods

### Clinical specimens

A total of 124 sputum samples were prospectively collected from random pulmonary tuberculosis suspects in Beijing Chest Hospital, these pulmonary tuberculosis suspects were continuously enrolled. After smear test, all samples were processed for BACTEC MGIT 960 culture and VereMTB Detection Kit test.

### Sample processing

Sputum samples were liquified and decontaminated with NaOH/NALC (N-Acetyl-L-Cysteine)-Na Citrate Solution (Mixture of 4% NaOH, 0.5% NALC and 2.9% Na Citrate) [5]. After centrifugation, the sediments were resuspended in 2 ml of 0.067 M Phosphate Buffer Solution (PBS, pH 7.0). Two aliquots were prepared to perform BACTEC MGIT 960 culture and VereMTB Detection Kit test respectively.

### DNA extraction

Sputum sediment from 1 ml aliquot was resuspended in 100 μL sterile water after centrifuge. The sediment was then incubated at 95°C-100°C for 20 minutes, followed by sonication in water bath sonicator for 15 minutes. Supernatant containing DNA was transferred into a new microcentrifuge tube.

### VereMTB Detection Kit

**Fig 1:**
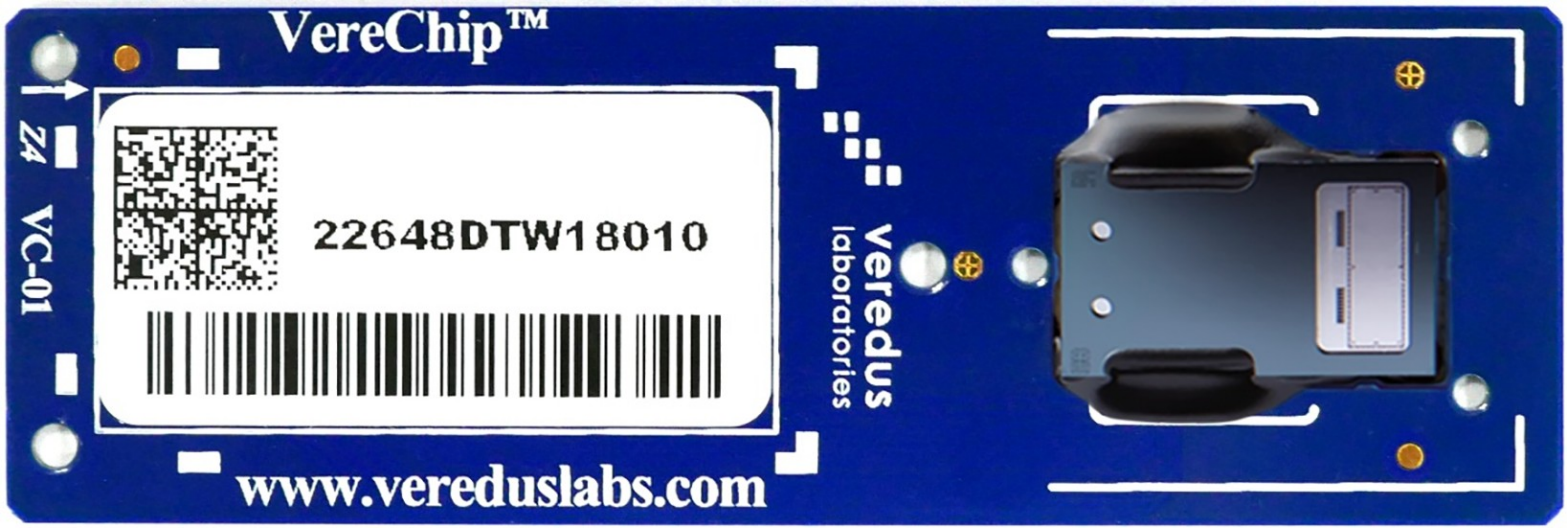
VereChip.

**Fig 2:**
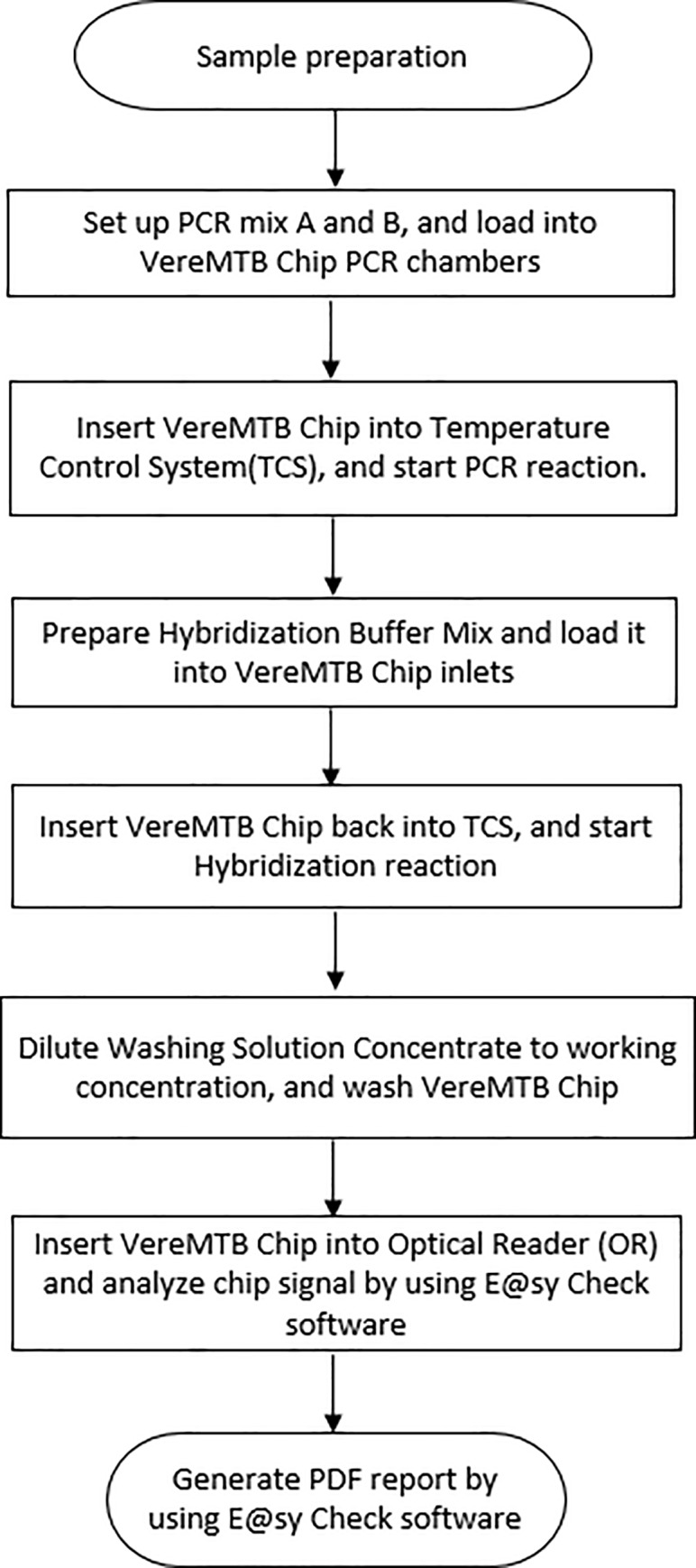
VereMTB test work flow.

VereMTB Detection Kit includes a single use disposable VereChip^™^ (Fig 1) which integrates a miniaturized PCR reactor, and a DNA microarray, as well as reagents and consumables necessary for nucleic acid amplification and DNA hybridization. The main steps for VereMTB test are shown in Fig 2.

Each DNA sample was added to two different PCR Master Mixes in two different tubes respectively. The prepared sample mix was loaded into two separate chambers on the VereChip through two inlets. The chip was sealed with clamps, placed into the Temperature Control Module (TCM) of the Temperature Control System (TCS). The PCR reaction was performed within the Chip chambers using the amplification protocol previously mentioned [4]. Upon completion of the PCR protocol, the hybridization buffer mix was loaded through the same inlets for PCR Master Mix loading. This pushed the PCR products out of the outlet to the microarray area for DNA hybridization. Chips were clamped and placed back into the TCM for the hybridization reaction. At completion of hybridization, the Chip was transferred into a 50 mL conical tube filled with wash buffer and centrifuged at 1500 x g for 2 minutes and dried in an empty tube using the same settings. After drying, a chip image was captured with the Optical Reader (Veredus Laboratories Pte Ltd) and the VerePLEX^TM^ software automatically performed the analysis generating a report based on the diagnostic rules fixed for target detection.

### BACTEC MGIT 960 culture and drug susceptibility study

0.5 ml sputum sediment aliquots were inoculated in BACTEC-MGIT 960 tubes (MGIT; Becton Dickinson) and incubated at 37°C in BACTEC MGIT960 system (Becton Dickinson). After the culture flashed positive, the susceptibility test to RIF (1 μg/m1) and INH (0.1 μg/m1) was performed according to the manufacturer’s protocol [5].

### Sequencing

All the culture positive strains were collected for DNA sequencing to identify *Mycobacterium* species related gene (*16S rRNA*) and drug resistance related gene mutation for RIF (*rpoB*) and INH (*katG and inhA*) at National TB Reference Laboratory (Table 1). The sequencing results were entered into the Basic Local Alignment Search Tool (BLAST, http://www.ncbi.nlm.nih.gov/BLAST), for the alignment with reference *Mycobacterium tuberculosis* strain H37Rv to identify the mutations in *rpoB*, *katG* and *inhA* gene.

**Table 1:** Primers used for sequencing to identify MTBC and detection of drug resistance related genes.

Gene	Primer pairs(5'-3')	Amplification length(bp)
*16S rRNA*	F: GGCCTAACCCTCGGGAGGGAG	440
	R: CCCGAGGCATATCGCAGCCTC	
*rpoB*	F: ACCGACGACATCGACCACTT	430
	R: GTACGGCGTTTCGATGAACC	
*katG*	F: AATCGATGGGCTTCAAGACG	500
	R: CTCGTAGCCGTACAGGATCTCG	
*inhA*	F: CCTCGCTGCCCAGAAAGGGA	248
	R: ATCCCCCGGTTTCCTCCGGT	

### Ethics statement

This study was reviewed and approved by the ethics review board of the Chinese Center for Disease Control and Prevention. All the specimens we used were useless sample from clinic laboratory after routine examination. The requirement to obtain individual informed consent was waived by the review board.

## Results

### The performance of VereMTB Detection Kit for MTBC and NTM detection

A total of 124 sputum samples were tested for the validation, comprising of 60 Acid-Fast Bacilli (AFB) smear-negative samples and 64 AFB smear-positive samples (Table 2). Fifty-one out of the 60 smear-negative samples were culture negative and the remaining 9 smear-negative samples were culture positive. These 9 samples were confirmed to have MTBC according to sequencing result of *16S rRNA*. All of the smear-positive samples were culture positive; 58 smear-positive samples were culture-positive for MTB and the remaining 6 were deemed as NTM based on *16S rRNA* sequencing. Using the VereMTB Detection Kit, 57 out of these 58 smear-positive MTBC samples were detected. 5 out of the 6 NTM samples were identified as *M*. *intracellulare* and one sample was determined to be *M*. *kansasii*, and this result was in accordance with *16S rRNA* sequencing (Table 2).

**Table 2:** Comparison of MGIT culture, 16s rRNA sequencing and VereMTB Detection Kit test result of 124 sputum samples.

	60 AFB smear-negative samples	64 AFB smear-positive samples	In Total
**BACTEC MGIT culture**	9 positive (9 MTBC)	64 positive (58 MTBC, 6 NTM)	67 MTBC, 6 NTM positive
**16s rRNA sequencing**	9 MTBC^a^	58 MTBC, 5 *M*. *intracellulare*, 1 *M*. *kansasii*	67 MTBC, 5 *M*. *intracellulare*, 1 *M*. *kansasii*
**VereMTB Detection Kit**	9 MTBC Detected^b^	57 MTBC Detected; 5 *M*. *intracellulare* Detected; 1 *M*. *kansasii* Detected.	66 MTBC Detected; 5 *M*. *intracellulare* Detected; 1 *M*. *kansasii* Detected.

^a^Sequencing was conducted for culture positive samples only.

^b^Eight out of these 9 samples were MGIT culture positive.

Refer to Table 3 for data comparison of MGIT cultured method and VereMTB Detection Kit for AFB smear negative samples, smear positive samples, and all samples. 9 out of 60 AFB smear negative samples were MTBC culture positive, and 8 out of these 9 samples were VereMTB MTBC positive. 1 AFB smear negative and cultured negative sample showed MTBC positive result by VereMTB detection kit. 58 out of 64 AFB smear positive samples were MTBC culture positive, and 57 out of these 58 samples were VereMTB MTBC positive. The remaining 6 were deemed as NTM based on *16S rRNA* sequencing (refer to Table 2). In total, out of the 124 samples, 67 were MTBC culture positive, and 65 out of these 67 samples were VereMTB MTBC positive.

**Table 3:** Test result of MGIT Culture method and VereMTB Detection Kit for MTBC detection.

		AFB Smear Negative		AFB Smear Positive		Total 124 samples	
		MGIT Culture		MGIT Culture		MGIT Culture	
		MTBC Positive	MTBC Negative	MTBC Positive	MTBC Negative	MTBC Positive	MTBC Negative
**VereMTB Detection Kit**	**MTBC Detected**	8	1	57	0	65	1
	**MTBC Not Detected**	1	50	1	6	2	56

Summary of the performance of VereMTB Detection Kit for detection of MTBC is shown in Table 4. The overall sensitivity of the assay was 97.0% (65/67). The sensitivity in smear-positive and culture-positive cases was 98.3% (57/58), whereas in smear-negative and culture-positive cases, it was 88.9% (8/9). The specificity for the diagnosis of MTBC was 98.3% (56/57) for VereMTB Detection Kit. The positive predictive value (PPV) of the assay was 98.5% (65/66) for all samples. For smear-positive cases, PPV was 100% (57/57), and this value was 88.9% (8/9) for smear-negative cases. The overall NPV for VereMTB Detection Kit was 96.6% (56/58).

**Table 4:** Diagnostic performance of VereMTB Detection Kit for detecting MTBC from sputum specimens.

	All culture-positive	Smear-positive and MGIT culture positive	Smear-negative and MGIT culture positive	No MTBC^a^
** **	**Sensitivity**			**Specificity**
**True No./ Total No.(%)**	65/67(97.0)	57/58(98.3)	8/9(88.9)	56/57(98.3)
**95%Cl**	89.6–99.6	90.8–100.0	51.8–99.7	90.6–100.0
	**PPV**			**NPV**
**True No./ Total No.(%)**	65/66(98.5)	57/57(100)	8/9(88.9)	56/58(96.6)
**95%Cl**	90.3–99.8	N.A.	53.1–98.3	87.7–99.1

^a^Comprises of MGIT culture negative and MGIT culture positive NTM samples

### The performance of VereMTB Detection Kit for detecting RIF and INH resistance

Drug susceptibility study for RIF and INH was performed using VereMTB Detection Kit and MGIT DST. 63 out of the 66 “MTBC detected” samples displayed valid result by VereMTB Detection Kit, and the remaining 3 samples were all smear negative samples which gave “indeterminate” result for drug susceptibility. Refer to Tables 5 & 6 for the details.

**Table 5:** Test result of MGIT DST and VereMTB Detection Kit for RIF susceptibility.

		MGIT DST	
		RIF Resistant	RIF Susceptible
VereMTB Detection Kit	RIF Resistant	12	3^b^
	RIF Susceptible	2^a^	46

^a^Both contained genotypic mutation in *rpoB* gene by sequencing, the mutations were H526L and H526Y which were consistent with VereMTB’s result.

^b^Two showed genotypic mutation in *rpoB* gene by sequencing, the mutations were H526Y and D516V. The third discordant strain has no mutation according to sequencing result, while the VereMTB detection kit showed S531L mutation.

**Table 6:** Test result of MGIT DST and VereMTB Detection Kit for INH susceptibility.

		MGIT DST	
		INH Resistant	INH Susceptible
VereMTB Detection Kit	INH Resistance	12	2^b^
	INH Susceptible	4^a^	45

^a^No genotypic mutation was detected in all these 4 samples by sequencing.

^b^One was confirmed to contain genotypic mutation in *katG* gene by sequencing, the mutation was S315T.

Out of the 63 samples with valid drug susceptibility result, 46 samples gave RIF susceptible result by both VereMTB Detection Kit and MGIT DST. Twelve of the samples determined as RIF resistant from MGIT DST was consistently determined as RIF resistant using VereMTB Detection Kit. Two specimens were, however, inconsistent with MGIT DST where these samples were identified as RIF susceptible by VereMTB Detection Kit. These were by sequencing results indicating genotypic mutation (H526L and H526Y) within the *rpoB* gene of both samples. Three MGIT DST RIF susceptible specimens were identified as RIF resistance by VereMTB Detection Kit, further DNA sequencing analysis revealed that 2 of which were confirmed to be genotypically resistant due to mutation (H526Y and D516V) in *rpoB* gene (Table 5).

57 samples gave consistent INH susceptibility result by using VereMTB Detection Kit and MGIT DST (12 INH resistant and 45 INH susceptible). Four phenotypically INH resistant strains exhibiting INH susceptibility by VereMTB Detection Kit were sequenced revealing that all samples carried no mutations in *katG* and *inhA* genes. Two samples determined to be INH resistant by VereMTB but susceptible to INH by MGIT DST was sequenced. One of these sequenced samples was confirmed to be genotypically resistant with mutation (S315T) of the *katG* gene (Table 6).

In terms of diagnostic accuracy for RIF and INH resistance when comparing to the MGIT DST, VereMTB Detection Kit showed good sensitivity (85.7% and 75.00% respectively) and excellent specificity (93.9% and 95.7% respectively) (Table 7).

**Table 7:** Diagnostic performance of VereMTB Detection Kit for detecting RIF and INH resistance.

Drug	Sensitivity	Specificity	PPV	NPV
**RIF**				
True No./Total.(%)	12/14(85.7)	46/49(93.9)	12/15(80.0)	46/48(95.8)
95%Cl	57.2–98.2	83.1–98.7	56.7–92.4	86.4–98.8
**INH**				
True No./Total No.(%)	12/16 (75.0)	45/47 (95.7)	12/14 (85.7)	45/49 (91.8)
95%Cl	47.6–92.7	85.5–99.5	60.0–96.0	82.8–96.3

## Discussion

Early and accurate diagnosis is critical to interrupt transmission of TB and MDR TB, especially in the absence of an effective vaccine [6]. This is the first evaluation of sputum specimens from pulmonary TB suspects in China using the VereMTB Detection Kit. Our results showed that theVereMTB Detection Kit can effectively diagnose MTBC, NTM and MDR-TB from sputum samples. Relative to the MGIT DST culture method, the turnaround time of VereMTB Detection Kit was sharply shortened from 15 days to 3 hours.

MTB Detection Kit showed good sensitivity (97.0%) and specificity (98.3%) for MTBC detection. However, 1 false positive result was observed which might be due to the carry over contamination from previous tested samples since it is a PCR based detection kit.

For drug resistance detection, VereMTB Detection Kit was able to give valid result for all smear positive and MTBC detected samples, some of the smear negative and MTBC detected samples (5/8), and showed good sensitivity (85.7% for RIF and 75% for INH) and excellent specificity (93.9% and 95.7% respectively). This kit provided “indeterminate” drug resistance result when MTBC was detected but the kit sensitivity was not enough to conclude drug susceptibility information.

Recent studies have shown that rapid liquid culture systems such as MGIT 960 and the proportion method with shorter incubation time (4 weeks) often fail to detect strains that are resistance to RIF, particularly strains contain specific mutations within *rpoB* gene codon 511 (such as L511P), codon 516 (such as D516Y), codon 526 (such as H526N, H526L and H526S), and codon 533 (such as L533P) [7,8]. Patients infected with MTBC with the above disputed *rpoB* mutations often fail treatment or relapse due to low-level resistance to RIF (minimum inhibitory concentration, MIC of 0.5–2.0 μg/ml) [9,10]. VereMTB Detection Kit is able to detect L511P mutation in *rpoB* gene, and is indicative of mutation within the *rpoB* hot-spot region (*rpoB* wild type probe missing), which provide low-level RIF resistance information to doctors. In this study, two samples carrying *rpoB* H526L mutation were RIF resistant according to MGIT DST result. One was detected by VereMTB chips (RIF resistant and *rpoB* wild type probe missing) and the other was not detected.

Mutations in *katG* gene and *inhA* gene are responsible for about 75% isoniazid-resistant isolates [11]. Drug resistance determination on the VereMTB Detection Kit interrogates mutations in these genes. The sensitivity of VereMTB Detection Kit for detection of INH resistance was 75.0%. The potential cause of this low performance could be due to mutations outside of the *katG* gene and *inhA* gene, not analyzed on the VereMTB Detection Kit. In this study, four INH phenotypically resistant samples showed INH susceptibility using VereMTB Detection Kit. Sequencing results revealed that all 4 samples carried no mutations in *katG* and *inhA* gene suggesting a potential mutation out of the VereMTB detection scope. According to recent research, the *oxyR-ahpC* gene region has been implicated as an indicator of the isoniazid resistance [12, 13]. Thus, this lower sensitivity for INH resistance was likely associated with the high prevalence of *oxyR-ahpC* region mutations within China, which is not detected by the VereMTB Detection Kit.

One unique feature of VereMTB Detection Kit is its ‘two in one’ capability in combining MTB drug resistance detection and common NTM detection. The assay successfully differentiated and identified both MTB and NTM, with the identified NTMs confirmed using sequencing. The performance of NTM detection has, however, not been rigorously tested due to the small test size of NTM in this study. The VereMTB Detection Kit covers a 14 NTMs including some of the common NTMs in China- *M*. *intracellulare*, *M*. *avium*, *M*. *abscessus/chelonae*, *M*. *kansasii* and *M*. *fortuitum* [14, 15]. With rising NTM infections in China [16] and cases of NTM pulmonary infection being misdiagnosed as pulmonary TB due to the similar clinical symptoms and current diagnostic criteria [17, 18], the VereMTB Detection Kit may be a useful tool for diagnostic and clinical use.

However, due to the limitation of number of probes the current Verechip microarray is able to fix (maximum 126 probes include hybridization control probes, PCR control probes and all detection probes, spotted in 3 replicates to avoid “ambitious” situation), VereMTB Detection Kit is only capable to detect MTBC, main NTM, and stain susceptibility to rifampicin and isoniazid. This assay has potential of detecting more resistance-conferring mutations, and strain susceptibility to more antibiotics if higher density VereChip platform is in operation.

We also realized the limitation of this study. It was just a preliminary validation study on new molecular assay and we just collected relatively small number sample specimens. The performance looks good and large scale sample evaluation studies across multiple communicable disease centers in order to further examine the robustness of this assay will be needed in the future.

## Conclusions

In conclusion, the VereMTB Detection Kit provides a much shorter turnaround time compared to conventional DST. According to our evaluation result, the VereMTB Detection Kit showed good sensitivity in detecting MTB and RIF and/or NIH resistance TB from sputum specimens, and it was also able to detect and identify NTM in the same test.

## Supporting information

S1 Dataset (XLSX)

## References

[1] World Health Organization, 2018. Global Tuberculosis Report. Available from: http://www.who.int/tb/publications/global_report/en/.

[2] Somoskovi A, Salfinger M. The race is on to shorten the turnaround time for diagnosis of multidrug-resistant tuberculosis. J Clin Microbiol. 2015;53(12): 3715–3718. 10.1128/JCM.02398-15 10.1128/JCM.02398-15 26378276 PMC4652131

[3] Lazzeri E, Santoro F, Oggioni MR, Iannelli F, Pozzi G. Novel primer-probe sets for detection and identification of mycobacteria by PCR-microarray assay. J Clin Microbiol. 2012;50(11): 3777–3779. 10.1128/JCM.02300-12 22972818 PMC3486262

[4] Cabibbe AM, Miotto P, Moure R, Alcaide F, Feuerriegel S, Pozzi G, et al Lab-on-chip-based platform for fast molecular diagnosis of multidrug-resistant tuberculosis. J Clin Microbiol. 2015;53(12): 3876–3880. 10.1128/JCM.01824-15 26246486 PMC4652106

[5] Use I. BACTECTM MGITTM 960 SIRE Kit for the antimycobacterial susceptibility testing of Mycobacterium tuberculosis. 1–18.

[6] Kik S V, Qin Z Z, Pai M. Optimal diagnosis: how early and improved diagnosis can help prevent TB transmission, Clinical Insights: Tuberculosis Prevention, Future Medicine Ltd, 2014, pp. 7–32.

[7] Van Deun A, Aung KJ, Bola V, Lebeke R, Hossain MA, de Rijk WB, et al Rifampin drug resistance tests for tuberculosis: challenging the gold standard. J Clin Microbiol. 2013;51:2633–40. 10.1128/JCM.00553-13 23761144 PMC3719626

[8] Rigouts L, Gumusboga M, de Rijk WB, Nduwamahoro E, Uwizeye C, de Jong B, et al Rifampin resistance missed in automated liquid culture system for Mycobacterium tuberculosis isolates with specific *rpoB* mutations. J Clin Microbiol. 2013;51:2641–5. 10.1128/JCM.02741-12 23761146 PMC3719602

[9] Van Ingen J, Aarnoutse R, de Vries G, Boeree MJ, van Soolingen D. Low-level rifampicin-resistant Mycobacterium tuberculosis strains raise a new therapeutic challenge. Int J Tuberc Lung Dis. 2011;15:990–2. 10.5588/ijtld.10.0127 21682979

[10] Van Deun A, Aung KJM, Hossain MA, de Rijk P, Gumusboga M, Rigouts L, et al Disputed *rpoB* mutations can frequently cause important rifampicin resistance among new tuberculosis patients. Int J Tuberc Lung Dis. 2015;19:185–90. 10.5588/ijtld.14.0651 25574917

[11] Seifert M, Catanzaro D, Catanzaro A, Rodwell T. Genetic mutations associated with isoniazid resistance in Mycobacterium tuberculosis: a systematic review. PloS One. 2015;10(3):e0119628 10.1371/journal.pone.0119628 . 25799046 PMC4370653

[12] Zhang Z, Lu J, Liu M, Wang Y, Qu G, Li H, et al Genotyping and molecular characteristics of multidrug-resistant Mycobacterium tuberculosis isolates from China. J Infect. 2015;70(4): 335–345. 10.1016/j.jinf.2014.11.008 . 25485999

[13] Almeida Da, Silva PE Palomino JC. Molecular basis and mechanisms of drug resistance in Mycobacterium tuberculosis: classical and new drugs. J Antimicrob Chemother. 2011;66(7): 1417–1430. 10.1093/jac/dkr173 . 21558086

[14] Jing H, Wang H, Wang Y, Deng Y, Li X, Liu Z, et al Prevalence of Nontuberculous Mycobacteria Infection, China, 2004–2009. Emerg Infect Dis. 2012;18(3): 527–528. 10.3201/eid1803.110175 22376989 PMC3309567

[15] Wang X, Li H, Jiang G, Zhao L, Ma Y, Javid B, et al Prevalence and drug resistance of nontuberculous mycobacteria, Northern China, 2008–2011. Emerg Infect Dis. 2014;20(7): 1252–1253. 10.3201/eid2007.131801 24959839 PMC4073877

[16] Wu J, Zhang Y, Li J, Lin S, Wang L, Jiang Y, et al Increase in nontuberculous mycobacteria isolated in Shanghai, China: results from a population-based study. PLoS One. 2014;9(10). 10.1371/journal.pone.0109736 25330201 PMC4199589

[17] Maiga M, Siddiqui S, Diallo S, Diarra B, Traoré B, Shea YR, et al Failure to recognize nontuberculous mycobacteria leads to misdiagnosis of chronic pulmonary tuberculosis. PLoS One. 2012;7(5). 10.1371/journal.pone.0036902 22615839 PMC3353983

[18] Xu K, Bi S, Ji Z, Hu H, Hu F, Zheng B, et al Distinguishing nontuberculous mycobacteria from multidrug-resistant Mycobacterium tuberculosis, China. Emerg Infect Dis. 2014;20(6):1060–1062. 10.3201/eid2006.130700 24856951 PMC4036753

